# Supplementary material for: Effects of virtual reality intervention dosage on gait performance in Parkinson’s disease: a systematic review and meta-analysis
Source: PeerJ. 2025 Nov 21;13:e20320. doi: 10.7717/peerj.20320 (PMC12642915; doi:10.7717/peerj.20320)
Supplement: Supplemental Information 2 [file peerj-13-20320-s002.docx]

**1. Rationale for Conducting This Meta-Analysis**

Gait impairment is a prominent and debilitating symptom in individuals with Parkinson’s disease (PD), contributing to reduced mobility, increased fall risk, and decreased quality of life. Virtual reality (VR) training has emerged as a rehabilitation approach aimed at improving gait performance in PD. However, existing randomized controlled trials (RCTs) have reported heterogeneous results, and there is variability in patient populations, intervention protocols, and outcome measures. Given the growing body of research, a systematic review and meta-analysis was warranted to update the evidence, quantify the effects of VR-based interventions on gait outcomes, and explore potential moderating factors such as age, disease duration, intervention duration, and session time. This study aims to provide a comprehensive synthesis to support evidence-based clinical decision-making in PD rehabilitation.

**2. Contribution to Knowledge Compared to Previously Published Reviews**

This meta-analysis advances the current understanding of VR-based interventions for gait improvement in PD through several methodological enhancements:

It includes thirteen RCTs published between 2015 and 2023, encompassing a total of 541 participants, thereby expanding the available evidence compared to prior reviews.

Subgroup analyses were conducted based on predefined factors (age, disease duration, intervention period, session time, and intervention frequency), offering more detailed insights into variables that may influence intervention effectiveness.

Sensitivity analyses were performed using a leave-one-out approach to assess the robustness of the findings, demonstrating the stability of the overall results.

Study quality was systematically assessed using the PEDro scale, with scores ranging from 6 to 8 (mean 6.77), reflecting generally moderate to high methodological quality.

The certainty of the evidence for the primary outcome (gait improvement) was evaluated using the GRADE framework and rated as high quality.

While sensitivity analyses and GRADE assessments are standard methodological practices, their rigorous application in this review enhances the transparency and credibility of the findings. Overall, this study provides an updated, detailed synthesis and identifies key factors moderating the efficacy of VR interventions, thereby offering practical implications for optimizing personalized rehabilitation strategies for individuals with PD.
